# Trialkoxysilane Grafting in Alcohols: A Simple Approach towards Modified Silica-Based Materials

**DOI:** 10.3390/molecules29194730

**Published:** 2024-10-07

**Authors:** Paola Marzullo, Vincenzo Campisciano, Leonarda Francesca Liotta, Francesca D’Anna, Francesco Giacalone, Michelangelo Gruttadauria

**Affiliations:** 1 Department of Biological, Chemical and Pharmaceutical Sciences and Technologies (STEBICEF), University of Palermo, Viale delle Scienze, 90128 Palermo, Italy; paola.marzullo@unipa.it (P.M.); vincenzo.campisciano@unipa.it (V.C.); francesca.danna@unipa.it (F.D.); francesco.giacalone@unipa.it (F.G.); 2 Sustainable Mobility Center (Centro Nazionale per la Mobilità Sostenibile—CNMS), Via Durando 39, 20158 Milano, Italy; 3 Institute for the Study of Nanostructured Materials (ISMN), National Research Council (CNR), Via Ugo La Malfa 153, 90146 Palermo, Italy; leonardafrancesca.liotta@cnr.it

**Keywords:** alcohols, Knoevenagel reaction, silica gel, silanes, supported quaternary ammonium salt

## Abstract

The grafting of trialkoxysilanes is the most common method for the surface functionalization of silica gel, and it is usually carried out in the presence of toluene or other solvents such as acetonitrile or acetone. Here, we replaced these solvents with alcohols to afford silica materials containing alkoxy groups linked to the silicon atom. The grafting of *N,N*-dimethyl-3-amino- or 3-amino-propyltrimethoxysilane was carried out in the presence of several alcohols containing an unsubstituted alkyl chain (C7 and C14), a PEG functionalized chain, or an amino-substituted chain (*N,N*-dimethylamino, pyridyl). Materials were characterized via solid-state ^13^C- and ^29^Si CPMAS NMR and thermogravimetric analysis to prove that alcohols are not “innocent” solvents but take part in the reaction and lead to [RSi(OR^1^)-(OSi)_2_] systems where the OR^1^ group proceeds from the alcohol used in the synthesis. As a proof of concept, we briefly studied the catalytic activity of some of these materials with the aim of showing how different modifications can influence the course of a selected reaction. Finally, a quaternary ammonium salt (QAS)-based silica was prepared containing both an alkyl-QAS and an alkoxy-QAS linked to silicon atoms. This could represent an interesting approach for the development of new antifouling-based materials and, overall, the described strategy could be useful for the preparation of new organosilica materials.

## 1. Introduction

There is a paramount interest in the development of hybrid materials with well-defined properties for application in a wide range of fields such as catalysis, electrochemistry and chromatography, but also used as nanoreactors, nanofillers, sensors and in drug release, with antibacterial and antifouling activity, as well as in optical, magnetic and electronic applications. Hybrid materials are largely based on inorganic–organic structures such as silica-modified organic materials or materials based on carbon nanoforms [1,2,3,4,5,6,7,8,9].

During the past years, we have been involved in research devoted in the development of silica and carbon nanoform-based materials, mainly for catalytic applications [10,11,12,13,14].

**Scheme 1:**
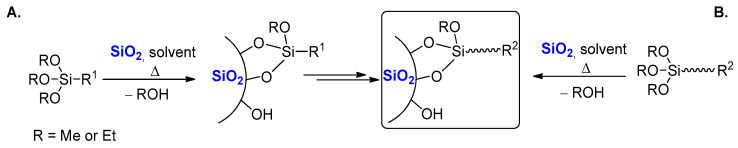
Synthetic approaches toward functionalized silica gel.

A quick Scopus search for silica hybrid materials yields more than 12,000 results. There are two main approaches for the obtainment of organic-modified silica-based materials: the direct sol-gel approach and the grafting of a proper trialkoxysilane (Scheme 1) [15]. In both cases, functionalized materials can be further modified to obtain the desired final product for the chosen purpose. Despite the huge number of papers on these subjects, there still is a great interest in developing new materials and new simple synthetic approaches toward them.

The simple grafting of trialkoxysilyl-modified molecules on silica gel remains the most common method for surface functionalization. The grafting mechanism involves, especially in the case of trichlorisilanes, hydrolysis to trisilanols followed by dehydration and cross-linking between silanes coupled with anchorage with surface silanols. However, this mechanism has been questioned and a direct reaction between trialkoxysilanes and surface silanols, via an addition-elimination type mechanism, has been proposed [16]. Generally, grafting is carried out by refluxing dry silica in the presence of the trimethoxy or triethoxysilane in solvents such as toluene or acetonitrile. The R^1^ group can be further modified to obtain the target material (Scheme 1, route A); the sequence can be reversed by first preparing the appropriate trialkoxysilane and then performing the final grafting procedure (Scheme 1, route B).

**Scheme 2:**
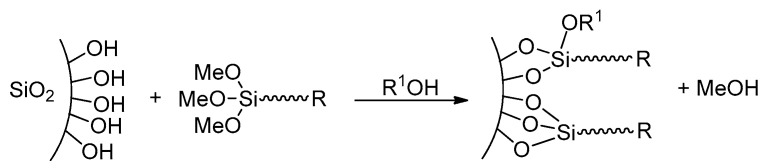
Grafting of trimethoxysilane on silica gel in alcohol.

These syntheses are so common that the examples are almost endless. Nevertheless, we envisaged the possibility of modifying this approach in a simple manner with the aim of obtaining new silica-based materials in a one-pot procedure, as depicted in Scheme 2. It should be noted that it is difficult to know the exact ratio of silane to alcohol in the final materials. Therefore, the reported chemical structures have to be intended as representative. This approach involves the use of a suitable alcohol as a solvent, which represents a greener alternative to classic organic solvents. In such a way, the final material can contain, in addition to the grafted silane via the T^3^ system [RSi(OSi)_3_], the grafted silane via T^2^ systems [RSi(OR^1^)-(OSi)_2_] in which the R^1^ group proceeds from the alcohol used in the synthesis and the excess of unreacted alcohol can be recovered in a pure form at the end of the reaction.

## 2. Results and Discussion

### 2.1. Grafting of (N,N-dimethyl-3-aminopropyl)trimethoxysilane in Heptanol as a Benchmark Reaction

As a benchmark reaction, we used (*N,N*-dimethyl-3-aminopropyl)trimethoxysilane and silica gel avoiding the use of toluene as a solvent for the grafting step. The first choice of the solvent was based on the GSK Solvent Sustainability Guide [17]. One of the most sustainable solvents is 1-heptanol; thus, we decided to use it bearing in mind our idea that it could react with silane to give a further modification of the final organic-silica material.

**Scheme 3:**
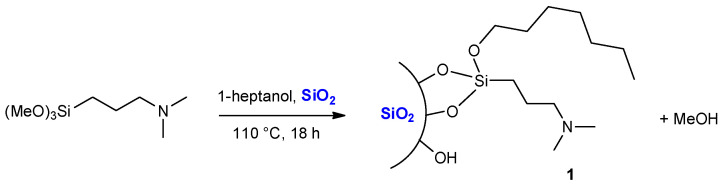
Preparation of the silica **1**.

The reaction was carried out using 2.0 g of dried silica, 2 mmol of silane and 30 mmol of 1-heptanol at 110 °C for 18 h. Then, the mixture was cooled at room temperature and filtered on a Gooch, washing with ethanol and a small amount of diethyl ether. The final material was then dried in the oven at 60 °C for 1 h (Scheme 3).

**Figure 1:**
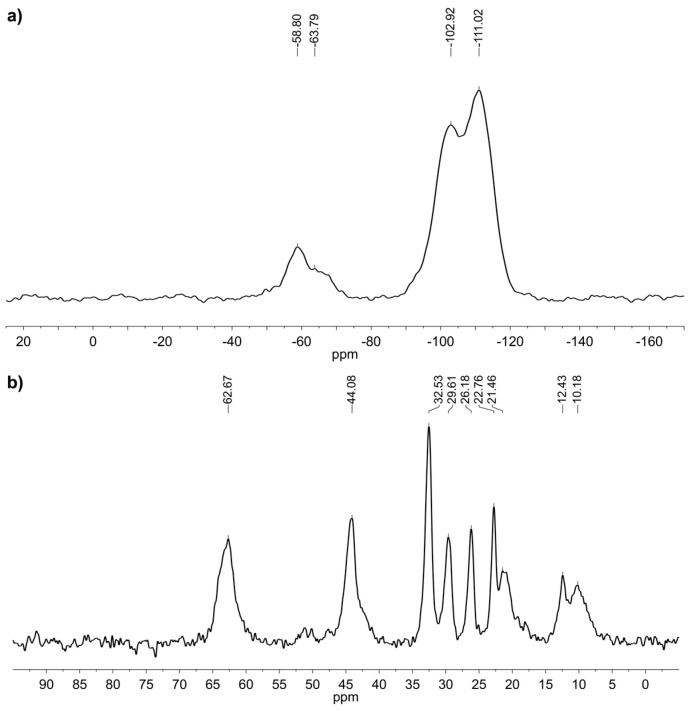
^29^Si (**a**) and ^13^C CPMAS NMR (**b**) spectra of the silica **1**.

The obtained material was characterized by solid-state ^29^Si and ^13^C CPMAS NMR (Figure 1). ^29^Si CPMAS NMR showed signals at ca. −111 and −103 ppm that can be assigned to Q^4^ [Si(OSi)_4_] and Q^3^ [Si(OSi)_3_OH] systems. The signal at ca. −64 ppm can be ascribed to T^3^ [RSi(OSi)_3_], whereas the more intense peak at ca. −59 ppm can be ascribed to T^2^ systems [RSi(OR^1^)-(OSi)_20_] [18,19]. To support our idea that the latter peak can be due to the heptyl moiety, we acquired the ^13^C CPMAS NMR spectrum (Figure 1b). This spectrum displays signals due to the (*N,N*-dimethyl)-3-aminopropyl chain. The peak at 44 ppm is ascribed to the *N,N*-dimethyl group, and the peak at 62 ppm can be ascribed to the methylene group linked to nitrogen atom while the peak at 10 ppm is due to the methylene group linked to the silicon atom. The additional peaks present can be assigned to the heptyl chain, and the peak at 12 ppm could be ascribed to the methyl group. Signals between 20 and 32 ppm can be assigned to the alkyl chain together with the methylene of the aminopropyl chain. The more deshielded signal in this group (32 ppm) could be related to the OCH_2_CH_2_ methylene, whereas the OCH_2_ methylene could resonate at 62 ppm. The very small peak at ca. 51 ppm could be due to a small amount of the methoxylsilyl group from the starting silane [5,10]. The NMR spectra confirm the one-pot functionalization of silica gel with both the silane and the alcohol.

**Figure 2:**
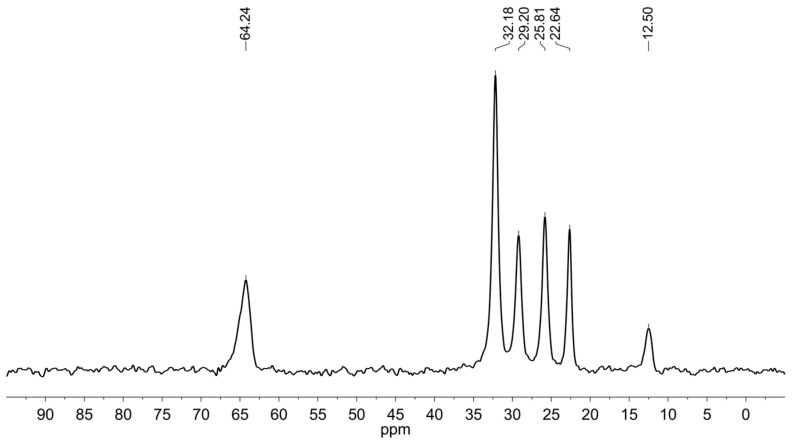
^13^C CPMAS NMR of the silica **2** material prepared by thermal treatment of silica gel with 1-heptanol.

With this evidence, we decided to carry out a thermal treatment of silica only with 1-heptanol to exclude any possible direct reaction with silanol groups. The ^13^C CPMAS NMR spectrum (Figure 2) showed the presence of heptanol, but the sharp nature of these signals (ca. 70 Hz) compared to the larger signals of the silica **1** indicated that heptanol is not covalently bound but adsorbed. This observation agrees with the covalent attachment to silica through the silicon atom of the silane when the reaction is carried out in the presence of the trimethoxysilane compound.

**Scheme 4:**
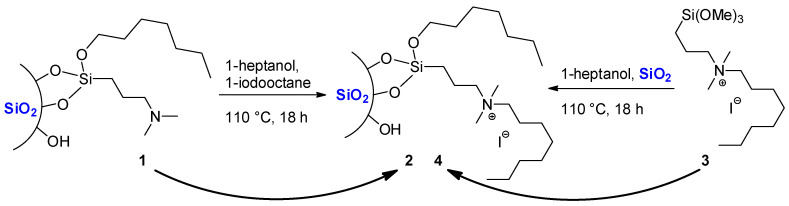
Preparation of the materials **2** and **4** by applying methods A and B depicted in Scheme 1.

As briefly described in Scheme 1, two different strategies can be followed to obtain the same material. Then, we explored the two approaches with the aim of obtaining a supported quaternized ammonium salt. Based on route A in Scheme 1, we modified the silica **1** by quaternization reaction of the tertiary amine with 1-iodooctane (silica **2**, Scheme 4). The reaction was carried out in 1-heptanol at 110 °C. Then, we followed route B in Scheme 1; to do so, we prepared the silane **3** by reaction between (*N,N*-dimethyl)-3-aminopropyltrimethoxysilane and 1-iodooctane and subsequently we carried out the grafting reaction of the silane **3** in 1-heptanol to give the material **4** (Scheme 4).

**Figure 3:**
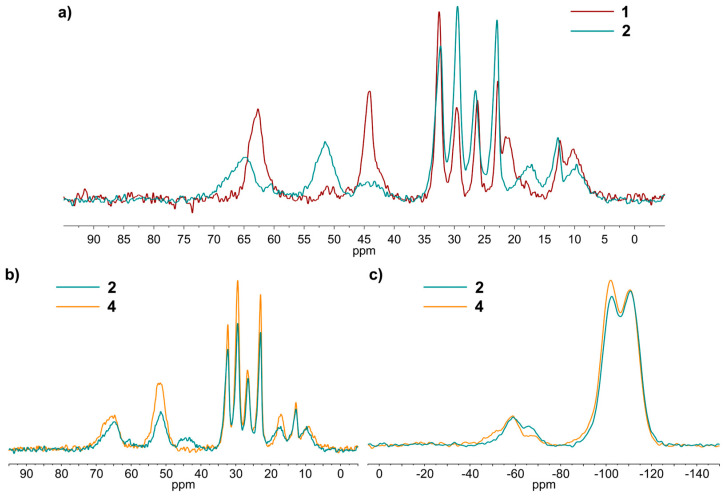
^13^C (**a**,**b**) and ^29^Si CPMAS NMR spectra (**c**) of the materials **1**, **2** and **4**.

In Figure 3a, the ^13^C CPMAS NMR of the silica **2** has been overlapped with the NMR spectrum of the silica **1**. From this overlapping, it is possible to see additional and more intense signals in the region between 10 and 35 ppm, whereas the *N,N*-dimethyl groups were deshielded at 51 ppm due to quaternization which could also be the reason for the less pronounced shift of the peak at about 65 ppm ascribed to the methylene group linked to the nitrogen atom. Quaternization could be incomplete because a small peak at ca. 44 ppm is still visible. In Figure 3b,c, the ^13^C and ^29^Si CPMAS NMR of the silica **2** overlapped with the NMR spectrum of the silica **4**, showing a complete superimposition. These data confirm that even when using a more complex silane such as the compound **3**, 1-heptanol acts both as a solvent and a reagent that is present in the final material. It should be noted that the Si-O(CH_2_)_6_CH_3_ moiety is stable after two steps of reaction.

**Figure 4:**
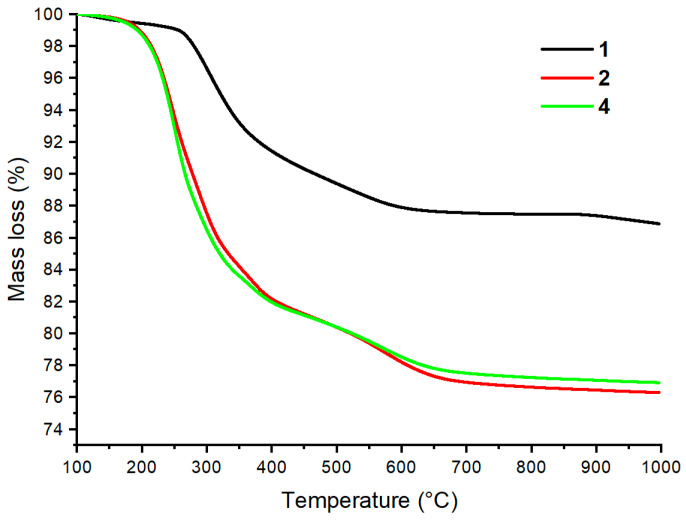
Thermogravimetric analysis of the materials **1**, **2**, and **4**.

TGA shows a very similar degradation profile and almost identical residue (Figure 4). This could mean that even by applying the post-functionalization technique, the amino sites in the silica **1** are well exposed and easily accessible.

### 2.2. Grafting of Trialkoxysilanes in the Presence of Different Alcohols

**Scheme 5:**
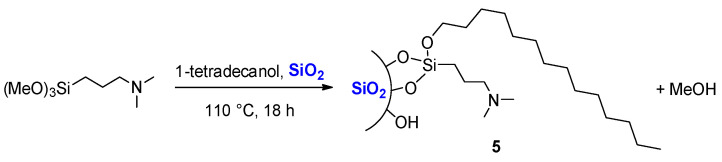
Preparation of the silica **5** material.

With the aim of further supporting this finding, we decided to use an alcohol with a longer alkyl chain, 1-tetradecanol. This alcohol is solid at room temperature, but it melts at 38 °C. Thus, we mixed silica gel and 1-tetradecanol and the mixture was heated at 60 °C until the latter compound melted, then trimethoxysilane was added and the mixture stirred at 110 °C. After 18 h, the mixture was cooled to room temperature and a solid was formed which was washed with ethanol and the mixture filtered (Scheme 5). The filtrate was evaporated and 1-tetradecanol was almost quantitatively recovered and its purity confirmed by ^1^H NMR. 

**Figure 5:**
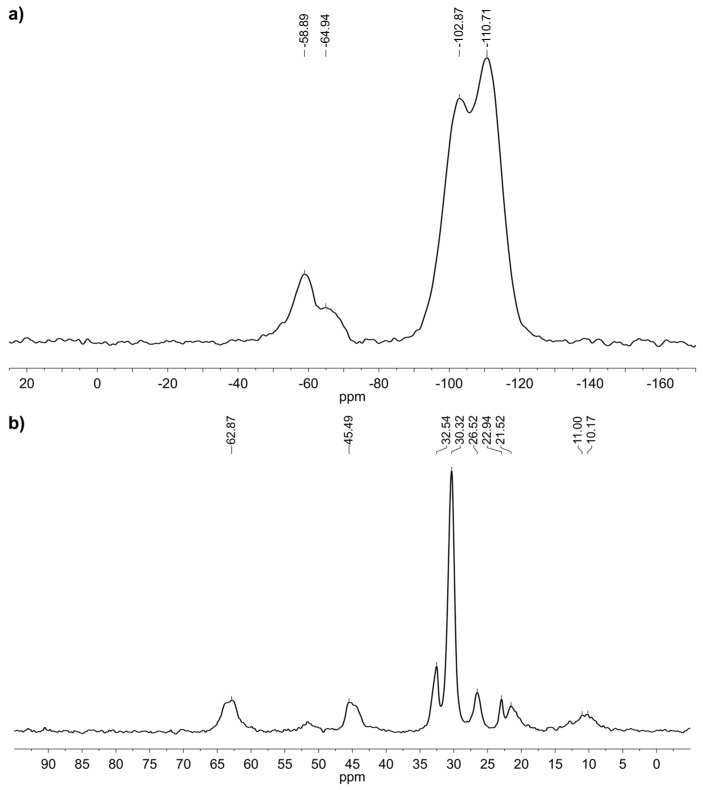
^29^Si (**a**) and ^13^C CPMAS NMR spectra (**b**) of the silica **5**.

The silica material was dried and characterized by solid-state NMR (Figure 5). ^29^Si CPMAS NMR showed signals that can be assigned to Q^4^ [Si(OSi)_4_] and Q^3^ [Si(OSi)_3_OH] systems and T^3^ [RSi(OSi)_3_] and T^2^ [RSi(OR^1^)-(OSi)_2_] systems with the latter mainly due to the attachment of 1-tetradecanol to the silicon atom of the silane. Again, the presence of the long alkyl chain was demonstrated by ^13^C CPMAS NMR. In addition to the signals already discussed, the strong signal at 30 ppm is ascribed to about nine carbon atoms of the alkyl chain.

**Figure 6:**
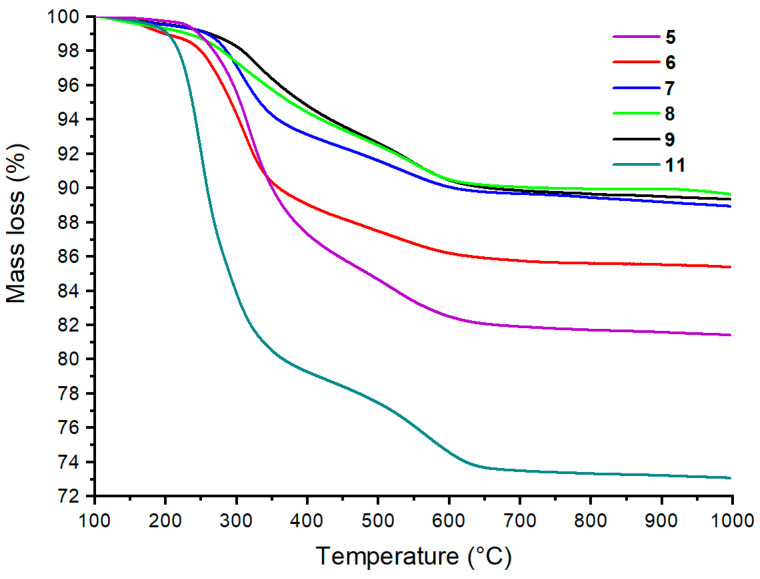
TGA under an air flow of the materials **5**–**9** and **11**.

Thermogravimetric analysis (TGA) carried out on these materials gave a similar content of adsorbed water and as expected, a higher organic content in the case of the silica **5** (18.6 wt%) and lower for the silica **1** (12.5 wt%) (Figure 6).

**Scheme 6:**
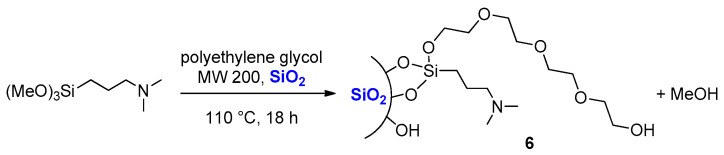
Preparation of the silica **6**.

**Figure 7:**
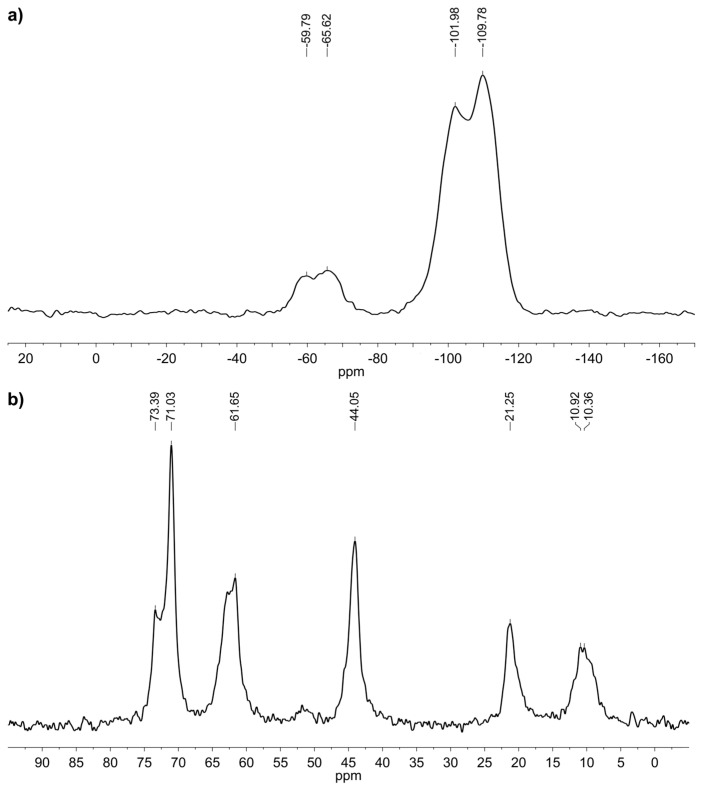
^29^Si (**a**) and ^13^C CPMAS NMR spectra (**b**) of the silica **6**.

The next step was to reverse the polarity of the alkyl chain. To do this, we used polyethylene glycol with an MW of 200 (average) (Scheme 6). Then, the collected material was characterized as usual. The ^29^Si CPMAS NMR spectrum showed the usual peaks due to T and Q systems present, though T^3^ and T^2^ peaks, differently from previous materials, appear similar in intensity. The ^13^C CPMAS NMR spectrum of the silica **6** showed peaks due to the polyethylene glycol at ca. 71 and 73 ppm. The dimethylaminopropyl moiety is clearly visible by peaks at ca. 61, 44, 21 and 10 ppm (Figure 7b). Again, the small peak at ca. 51 ppm could be ascribed to the presence of the residual methoxysilyl group.

TGA of the silica **6** allowed us to estimate an organic content of ca. 14.6 wt% (Figure 6).

**Scheme 7:**
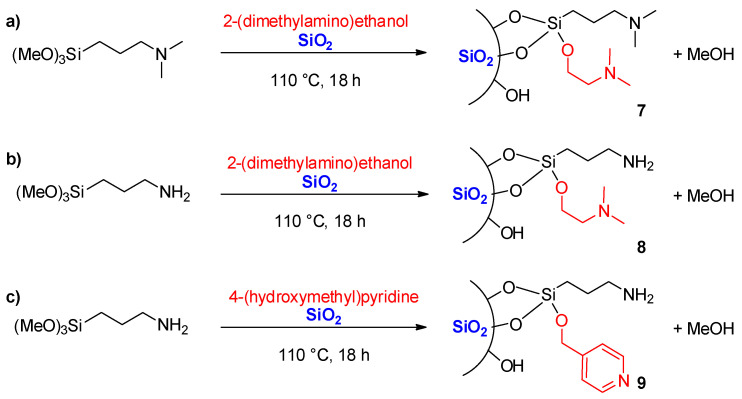
Preparation of the materials **7** (**a**), **8** (**b**) and **9** (**c**).

This synthetic methodology potentially has some interesting features: (a) the silane can be modified in a one-pot fashion; (b) if a more expensive alcohol is used, it can be easily recovered at the end of the reaction; (c) by using the suitable alcohol, a fine-tuning of the hydrophobicity/hydrophilicity of the modified silane linked at the surface of silica can be achieved. In the latter case, this could be useful if the material must be used for catalytic as well as for other purposes, such as antimicrobial activity. Furthermore, by using a functionalized alcohol, an additional functional group can be added. We then envisioned the possibility of developing materials with two functional groups bonded to the same silicon atom, thus being close to each other. In order to reach this aim, we carried out a reaction by using the same silane, (*N,N*-dimethyl-3-aminopropyl)trimethoxysilane, and 2-(dimethylamino)ethanol (Scheme 7a). Unreacted 2-(dimethylamino)ethanol was easily recovered. 

**Figure 8:**
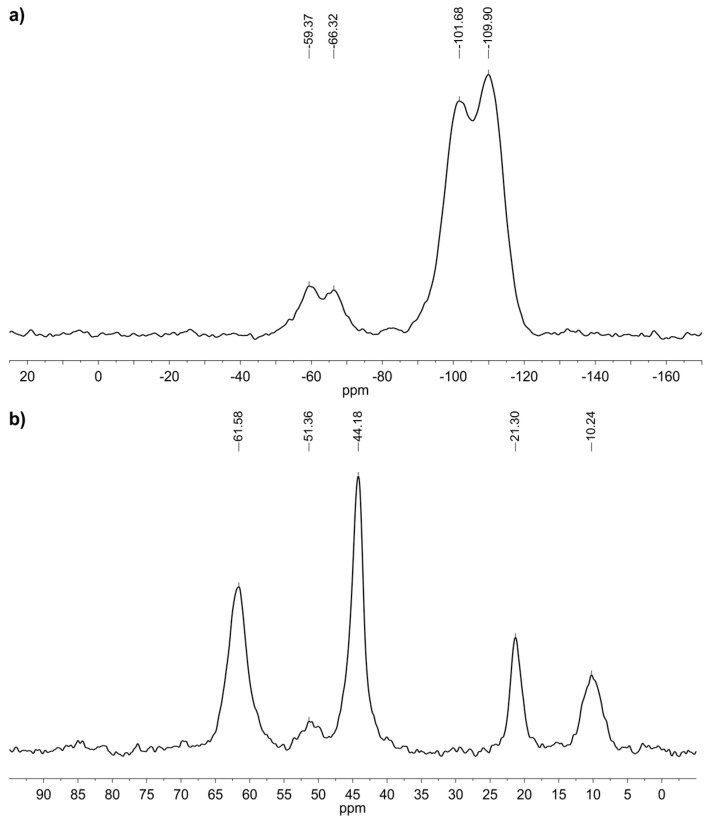
^29^Si (**a**) and ^13^C CPMAS NMR spectra (**b**) of the silica **7**.

The ^29^Si CPMAS NMR spectrum showed the usual peaks due to T and Q systems. The ^13^C CPMAS NMR spectrum shows the presence of both dimethylamino groups. The propyl chain is, as usual, clearly visible by the peaks at 10 and 21 ppm. On the other hand, the peak at 51 ppm could be ascribed to a small amount of the methoxy group of the starting silane (Figure 8).

We decided to explore the possibility of introducing two different amino moieties, a primary and a tertiary amine. In order to introduce a primary amino function, we used 3-aminopropyl-trimethoxysilane while as a tertiary amine, both aliphatic and aromatic, we used 2-(dimethylamino)ethanol or 4-(hydroxymethyl)pyridine (Scheme 7b,c).

**Figure 9:**
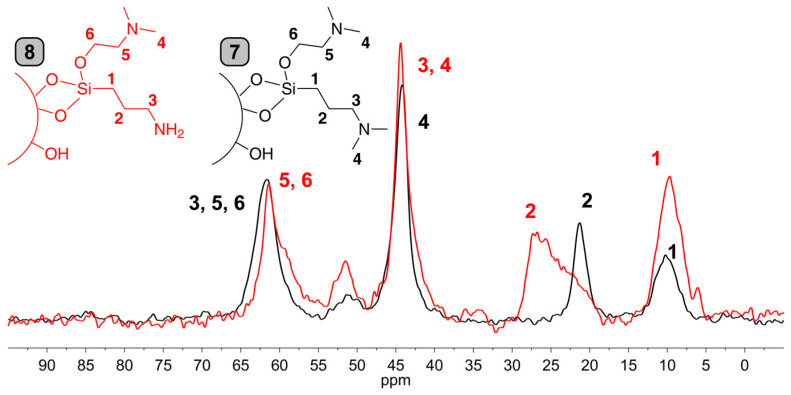
^13^C CPMAS NMR spectra of the materials **7** and **8**.

The overlapped ^13^C CPMAS NMR spectra of the materials **7** and **8** are shown in Figure 9. The main difference between the two spectra lies in the carbon atoms 2 and 3 of the silica **8**, respectively, resonating in a higher and lower field region with respect to the silica **7**. On the contrary, no remarkable differences were detected in the ^29^Si CPMAS NMR spectra of the silica **7** and the silica **8**.

**Figure 10:**
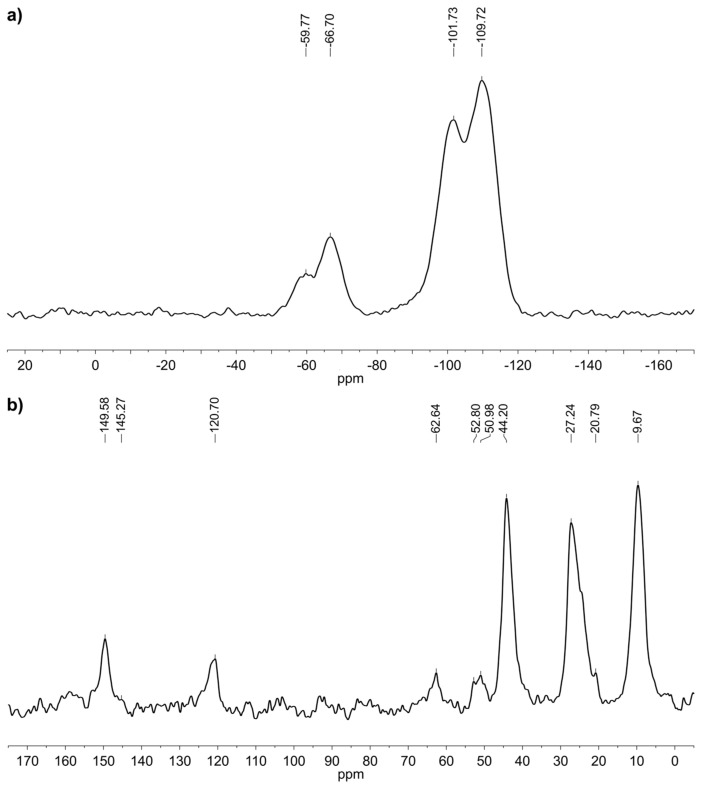
^29^Si (**a**) and ^13^C CPMAS NMR spectra (**b**) of the silica **9**.

The reaction in the presence of 3-aminopropyl-trimethoxysilane and 4-(hydroxymethyl)pyridine in Scheme 7c was carried out by mixing the latter compound with silica and heating at 60 °C until the pyridine melted. The silane was then added to the resulting silica suspension. The mixture was then heated at 100 °C for 15 h. After cooling at room temperature, a pale-yellow solid was formed which was washed with ethanol and the insoluble modified silica filtered. The washed solid was dried at 60 °C for 1 h, whereas the filtrate was evaporated to recover the unreacted 4-(hydroxymethyl)pyridine. The ^29^Si CPMAS NMR spectrum showed a higher T^3^ peak compared to T^2^, probably associated with a lower alcohol functionalization. Nevertheless, the ^13^C CPMAS NMR spectrum showed the presence of expected functionalization. Peaks at 150 and 120 ppm are related to the pyridine ring, while the peak at 62 ppm is due to the OCH_2_ methylene (Figure 10).

Thermogravimetric analysis of the materials **7**, **8** and **9** (Figure 6) indicated a very similar loading irrespective of the type of silane and alcohol used as well as the relative quantity of reactants with respect to silica (see the Experimental Section). The higher organic loading of the material **6** is due to the higher MW of the PEG compared to other alcohols.

### 2.3. Catalytic Activity in Knoevenagel Reaction

**Scheme 8:**
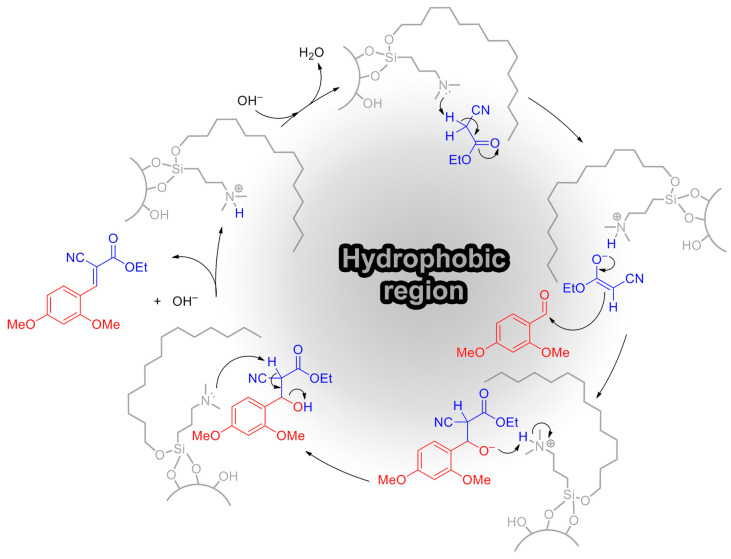
Catalytic mechanism for the synthesis of the Knoevenagel product in the presence of the silica **5**.

As a proof of concept, we briefly investigated the catalytic activity of some of these materials in order to show how the different modifications may influence the course of a selected reaction. To pursue this goal, we used the amine-catalyzed Knoevenagel reaction between 2,4-dimethoxybenzaldehyde and ethyl cyanoacetate as a benchmark reaction. The reaction was carried out in water at 30 °C. The use of water as a reaction medium was dictated by the fact that it is a green solvent as well as by the possibility of studying the different nature of the modified surface of the silica gel. We started our investigation by using the more hydrophobic modified silica **5** possessing the tetradecanyl moiety. The reaction was carried out in the presence of 32 mg of catalyst; after 1 h, a quantitative amount of the final compound was isolated (Table 1, Entry 1). An almost quantitative yield was also obtained in the presence of 15 mg of catalyst by doubling the reaction time (Entry 2). A lower yield was obtained when a higher amount of water was used (Entry 3). The use of the silica-based dimethylamine **1** possessing the heptanyl moiety gave a lower yield in comparison with the more hydrophobic material under similar reaction conditions (Entries 4 vs. 2). Then, we carried out the reaction in the presence of the PEG-modified material **6** at similar loading. We carried out three reactions under the same conditions reported in Entries 1–3. As can be seen from the comparison, in each of these cases the reactions always provided much lower yields (Entries 5–7). Material **7** possessing two *N,N*-dimethylamino groups gave a low yield too (Entry 8), again demonstrating that the role played by the hydrophobicity imparted by the chain outweighs the presence of an additional catalytic amine group. In particular, the higher catalytic activity of the silica **5**, in agreement with hydrophobically driven reactions [20,21], could be due to the generation of a hydrophobic region with a high affinity for reactants, which are forced to react in close proximity to the basic sites (Scheme 8).

It is well-known that alkoxysilanes undergo hydrolysis and reactions strongly depend on the structure of the alkoxy group: the larger the alkoxy group on the silane, the slower the hydrolysis rate [22,23].

**Figure 11:**
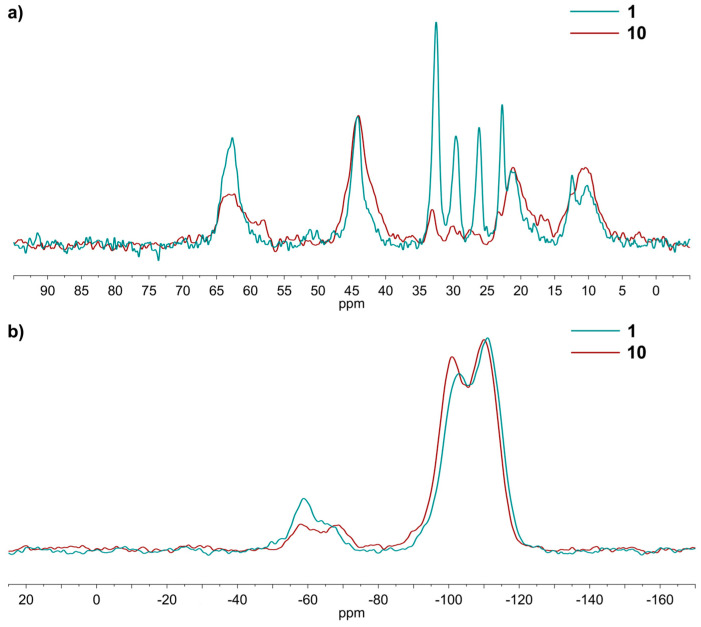
^13^C (**a**) and ^29^Si CPMAS NMR spectra (**b**) of the silica **1** and the silica **10**.

With the aim of obtaining an insight on the possible hydrolysis of the alkoxy group linked to the silicon atom, we treated the silica **1** (250 mg) with water (5 mL) at 25 °C for 19 h. The obtained material **10** was characterized via ^13^C and ^29^Si CPMAS NMR (Figure 11). ^13^C NMR revealed a decrease in signals related to the alkoxy chain. The ^29^Si CPMAS NMR spectrum showed no changes about signals related to Q^4^ and Q^3^ systems as well as signal at ca. −64 ppm, ascribed to the T^3^ system [RSi(OSi)_3_], whereas a decrease in the intensity of the peak at ca. −59 ppm [T^2^ systems RSi(OR^1^)-(OSi)_2_] was observed. This finding may have several consequences. First, the different behavior in Knoevenagel reaction could also be attributed to the different hydrolysis rate of the alkoxy group. Second, if an appropriate alkoxy group with tunable hydrolysis properties is present, a material with peculiar activity could be prepared.

### 2.4. Dual-Functional Silica-Based Materials for Antifouling Applications

**Scheme 9:**
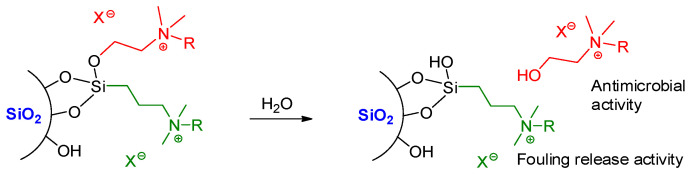
Possible dual antifouling activity.

We envisaged the possibility of synthesizing silica-based materials with linked quaternary ammonium salts (QASs) possessing an additional QAS unit linked through Si-O, although to a lesser extent with respect to the main QAS (Scheme 9). The purpose behind this decision is the possibility of developing silica materials with dual antifouling activity. On the one hand, the ammonium salt bound to the silicon atom through a strong Si-C bond confers fouling release or contact killing activity mediated by the hydrophobic alkyl chain. On the other hand, the controlled release of the second ammonium salt through the hydrolysis of the Si-O bond confers antifouling activity by releasing a biocide [24].

The hydrolysis of the Si-OR bond would release a choline-based ammonium salt that possesses a good antimicrobial activity and low cytotoxic properties when long alkyl chains are present [25]. This approach is described in Scheme 9 in which the R group should possess more than 10 carbon atoms. Tunable hydrolysis of the Si-O bond can release the choline-based salt with antimicrobial activity, while the quaternary ammonium salt linked via a more stable Si-C bond can impart fouling-releasing activity.

**Scheme 10:**
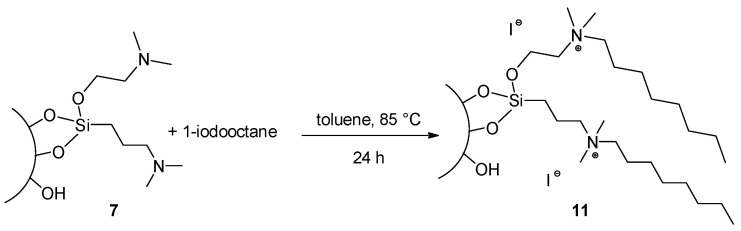
Preparation of the silica **11**.

Therefore, we treated the silica **7** with 1-iodooctane at 85 °C in toluene (Scheme 10).

**Figure 12:**
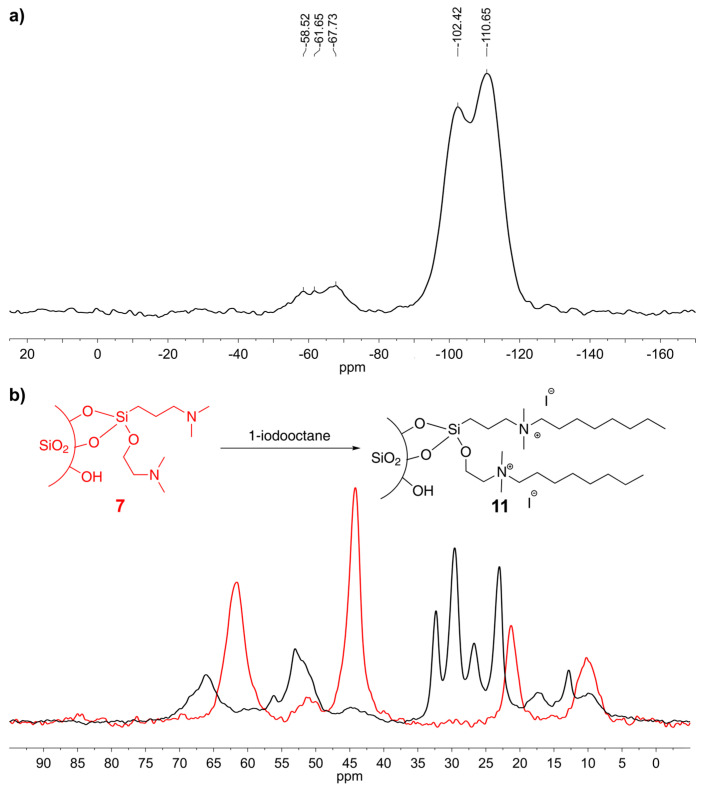
^29^Si CPMAS NMR spectrum of the silica **11** (**a**) and overlapped ^13^C CPMAS NMR spectra (**b**) of the silica **7** and the silica **11**.

This reaction was carried out twice, using a different amount of 1-iodooctane. However, no increased amount of the recovered final silica was obtained when a higher amount of 1-iodooctane was used. TGA shows an increase in organic loading (Figure 6), whereas ^13^C-NMR (Figure 12b) shows the presence of the alkyl chain as well as the deshielding of methyl and methylene groups linked to nitrogen atoms [26]. Methyl groups linked to the nitrogen atom at ca. 45 ppm were deshielded at ca. 53 ppm, as well as methylene groups linked to the nitrogen atom (from ca. 62 ppm to ca. 66 ppm), whereas methylene *C*H_2_OSi was shielded from ca. 62 ppm to ca. 55 ppm.

## 3. Materials and Methods

### 3.1. Spectroscopic and Analytical Methods

Chemicals and solvents were purchased from commercial suppliers to be used without further purification. (*N,N*-dimethyl-3-aminopropyl)trimethoxysilane, 3-aminopropyltrimethoxysilane, 1-iodooctane, 2-(dimethylamino)ethanol and ethyl cyanoacetate were purchased from Fluorochem (Hadfield, UK); 1-heptanol, 1-tetradecanol, polyethylene glycol (MW 200), 4-(hydroxymethyl)pyridine and 2,4-dimethoxybenzaldehyde were purchased from Sigma-Aldrich (Burlington, MA, US). All solvents (ACS grade) were purchased from VWR International (Milan, IT).

Silica gel 60 (0.040–0.063 mm) (Merk, Milan, IT) with a surface area of 480–540 m^2^/g (BET) was dried under vacuum for 5 h at 120 °C and used directly.

Thermogravimetric analysis (TGA) measurements were carried out under oxygen flow from 100 to 1000 °C with a heating rate of 10 °C min^−1^ in a Mettler Toledo TGA STAR (Milan, IT).

^29^Si Cross-Polarization Magic Angle Spinning (CPMAS) NMR spectra were acquired on a Bruker (Billerica, MA, US) Advance II 400 MHz (9.4 T) spectrometer operating at 79.5 MHz with a MAS rate of 6 kHz, 90° pulse on 1 H of 5.1 µs, a delay time of 2 s and a contact time of 8 ms.

^13^C Cross-Polarization Magic Angle Spinning (CPMAS) NMR spectra were acquired on a Bruker (Billerica, MA, USA) Advance II 400 MHz (9.4 T) spectrometer operating at 100.6 MHz with a MAS rate of 6 kHz, a delay time of 3 s and a contact time of 2 ms. The Hartman Hahn condition was optimized using adamantane as the standard.

### 3.2. Preparation of Silica ***1***

To a solution of (*N,N*-dimethyl-3-aminopropyl)trimethoxysilane (414.68 mg, 2 mmol) in 1-heptanol (4.25 mL, 30 mmol), dry silica gel was added (2.0 g) and the mixture stirred at 110 °C for 18 h. After cooling at room temperature, the silica was filtered through a Gooch filter and washed with ethanol and diethyl ether. The obtained material was dried in an oven to give 2.346 g of modified silica.

### 3.3. Preparation of Silica ***2***

1-Iodooctane (180.5 μL, 1.0 mmol) and 1-heptanol (2.12 mL, 15 mmol) were added to 500 mg of the silica **1** and the mixture was refluxed at 110 °C for 18 h. After cooling at room temperature, the silica was filtered through a Gooch filter and washed with ethanol and diethyl ether. The obtained material was dried in an oven to give 551 mg of modified silica.

### 3.4. Preparation of Silica ***4***

*N,N*-dimethyl-*N*-(3-(trimethoxysilyl)propyl)octan-1-aminium iodide **3** was prepared by reaction between (*N,N*-dimethyl-3-aminopropyl)trimethoxysilane (1.09 mL, 5 mmol) and 1-iodooctane (903 μL, 5 mmol) in toluene (4 mL) at 90 °C for 20 h. After this time, solvent was removed under reduced pressure until a pale-yellow oil was obtained. ^1^H-NMR (CDCl_3_) δ: 0.70 (t, J = 7.6 Hz, 2 H), 0.87 (t, J = 6.8 Hz, 3 H), 1.23–1.40 (m, 10 H), 1.70–1.85 (m, 4 H), 3.36 (s, 6 H), 3.45–3.55 (m, 4 H), 3.57 (s, 9 H). The obtained silane (895 mg, 2 mmol) and 1-heptanol (4.25 mL, 30 mmol) were added to dry silica gel (2 g) and the mixture stirred at 110 °C for 18 h. After cooling at room temperature, the silica was filtered through a Gooch filter and washed with ethanol and diethyl ether. The obtained material was dried in an oven to give 2.65 g of modified silica.

### 3.5. Preparation of Silica ***5***

In a round-bottom flask, 1-tetradecanol (4.29 g, 20 mmol) and dry silica gel (2.0 g) were added. The mixture was gently heated at 60 °C until the alcohol melted, then (*N,N*-dimethyl-3-aminopropyl)trimethoxysilane (414.68 mg, 2 mmol) was added and the mixture was stirred at 110 °C for 18 h. After cooling at room temperature, the alcohol solidified and it was dissolved by adding ethanol, and the silica was filtered through a Gooch filter and washed with more ethanol and diethyl ether. The obtained material was dried in an oven to give 2.50 g of modified silica. The ethanol solution was concentrated under reduced pressure, and unreacted pure 1-tetradecanol (by ^1^H-NMR) was recovered (93%).

### 3.6. Preparation of Silica ***6***

To a solution of (*N,N*-dimethyl-3-aminopropyl)trimethoxysilane (207.34 mg, 1 mmol) in polyethylene glycol (MW 200 ca. 3 g, 15 mmol), dry silica gel was added (1.0 g) and the mixture stirred at 110 °C for 18 h. After cooling at room temperature, the silica was filtered through a Gooch filter and washed with ethanol and diethyl ether. The obtained material was dried in an oven to give 1.212 g of modified silica. The ethanol solution was concentrated under reduced pressure, and unreacted PEG was recovered (96%).

### 3.7. Preparation of Silica ***7***

To a mixture of (*N,N*-dimethyl-3-aminopropyl)trimethoxysilane (414.7 mg, 2 mmol) and 2-(dimethylamino)ethanol (3.02 mL, 30 mmol), dry silica gel was added (2 g) and the mixture stirred at 110 °C for 18 h. After cooling at room temperature, the silica was filtered through a Gooch filter and washed with ethanol and diethyl ether. The obtained material was dried in an oven to give 2.344 g of modified silica. The ethanol solution was concentrated under reduced pressure, and unreacted 2-(dimethylamino)ethanol was recovered (95%).

### 3.8. Preparation of Silica ***8***

To a mixture of (3-aminopropyl)trimethoxysilane (717.2 mg, 4 mmol) and 2-(dimethylamino)ethanol (6.04 mL, 60 mmol), dry silica gel was added (2 g) and the mixture stirred at 110 °C for 18 h. After cooling at room temperature, the silica was filtered through a Gooch filter and washed with ethanol and diethyl ether. The obtained material was dried in an oven to give 2.430 g of modified silica. The ethanol solution was concentrated under reduced pressure, and unreacted 2-(dimethylamino)ethanol was recovered (95%).

### 3.9. Preparation of Silica ***9***

In a round-bottom flask, 4-(hydroxymethyl)pyridine (1.64 g, 15 mmol) and dry silica gel (0.5 g) were added. The mixture was gently heated at 60 °C until the alcohol melted, then (3-aminopropyl)trimethoxysilane (174.5 μL, 1 mmol) was added and the mixture was stirred at 110 °C for 15 h. After cooling at room temperature, the alcohol solidified and it was dissolved by adding ethanol and the silica was filtered through a Gooch filter and washed with more ethanol and diethyl ether. The obtained material was dried in an oven to give 0.6 g of modified silica. The ethanol solution was concentrated under reduced pressure, and unreacted pure 4-(hydroxymethyl)pyridine (by ^1^H-NMR) was recovered (97%).

### 3.10. Preparation of Silica ***11***

Silica **7** (400 mg) was suspended in toluene (3 mL), then 1-iodooctane (240 μL, 1.33 mmol) was added and the mixture heated at 85 °C for 24 h. After cooling at room temperature, the silica was filtered through a Gooch filter and washed with diethyl ether. The obtained material was dried in an oven to give 0.46 g of modified silica. A second preparation carried out in the presence of a higher quantity of 1-iodooctane (4 mmol) gave almost the same amount of final silica.

### 3.11. Typical Knoevenagel Reaction

To a suspension of 2,4-dimethoxybenzaldehyde (1 mmol) and modified silica (see Table 1) in water (1 or 3 mL), ethyl cyanoacetate (1.1 mmol) was added and the mixture was stirred at 30 °C for the indicated time. Then, dichloromethane was added and the organic phase was extracted. The final product was isolated using a short silica pad and identified by ^1^H NMR.

## 4. Conclusions

In conclusion, we have reported a simple methodology for the grafting of trimethoxysilanes onto the surface of silica gel using alcohols as the reaction medium. Usually grafting procedures are carried out in solvents such as toluene or acetonitrile, or in solvent-less conditions. When alcohols are used, they do not behave as “innocent” solvents but take part in the reaction and lead to [RSi(OR^1^)-(OSi)_2_] systems where the OR^1^ group proceeds from the alcohol used in the synthesis.

Alcohols containing an unsubstituted alkyl chain (C7 and C14), a PEG functionalized chain (MW ca. 200), or an amino-substituted chain (*N,N*-dimethylamino, pyridyl) were used for the grafting of (*N,N*-dimethyl-3-amino)- or 3-amino-propyltrimethoxysilane.

The materials were characterized via solid-state ^13^C- and ^29^Si CPMAS NMR and thermogravimetric analysis. The different nature (hydrophilic or hydrophobic) of the alcohol may impart a different behavior to the final material due to the exposure to the surface of silica of the alcohol moiety -OR^1^. Indeed, such a behavior was observed by conducting some Knoevenagel reactions between 2,4-dimethoxybenzaldehyde and ethyl cyanoacetate in water. In the presence of the silica **5** possessing a C14 residue, the more hydrophobic the surface, the more active the catalyst. This is in agreement with hydrophobically driven reactions [20,21].

This research opens new possibilities; first, by choosing the suitable alcohol, fine-tuning of the nature of silica surface can be achieved. Moreover, it should be taken into consideration that an excess of unreacted alcohol is easily recovered in pure form at the end of the reaction by simple removal of the solvent under reduced pressure. Second, by choosing a suitable alcohol, a quaternary ammonium salt (QAS)-based silica can be prepared containing both an alkyl-QAS and an alkoxy-QAS linked to silicon atoms.

To verify this hypothesis, we prepared the silica **11**. This strategy represents an interesting approach for the development of new materials possessing potential antibiofilm activity.

Indeed, as an ongoing application of this strategy, we are currently developing new silica materials, with a high surface area, to be modified with both an alkyl-QAS and an alkoxy-QAS linked to silicon atoms; then, these materials will be tested for antibiofilm formation.

## Figures and Tables

**Table 1:** Knoevenagel reaction between 2,4-dimethoxybenzaldehyde and ethyl cyanoacetate.

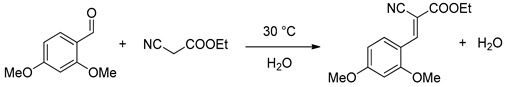					
Entry	Catalyst	Cat. Amount (mg)	Time (h)	Water (mL)	Yield (%)
entry 1	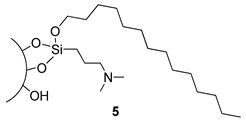	32	1	1	>99
entry 2		15	2	1	95
entry 3		15	2	3	61
entry 4	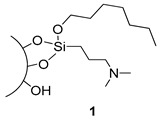	15	2	1	49
entry 5	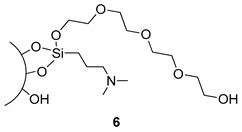	33.5	1	1	59
entry 6		16	2	1	24
entry 7		16	2	3	28
entry 8	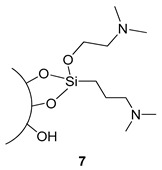	15	2	1	35

Reaction conditions: aldehyde (1 mmol), ethyl cyanoacetate (1.1 mmol), H_2_O (1 or 3 mL). Blank test: no reaction.

## Data Availability

Inquiries can be directed to the corresponding author/s.
